# Kawasaki disease shock syndrome with extreme leukemoid reaction mimicking hematologic malignancy in an IVIG-resistant child: A case report

**DOI:** 10.1097/MD.0000000000045845

**Published:** 2025-11-07

**Authors:** Lingke Liu, Wenjie Xuan, Yana Wang, Nan Tu, Xiaoxian Wang, Boting Pan

**Affiliations:** a Department of Pediatrics, The Affiliated Hospital of Shaoxing University, Shaoxing, PR China; b Department of Pediatrics, Shaoxing People’s Hospital (Shaoxing Hospital, Zhejiang University School of Medicine), Shaoxing, PR China.

**Keywords:** corticosteroids, IVIG resistance, Kawasaki disease shock syndrome, leukemoid reaction, pediatric vasculitis, thromboprophylaxis

## Abstract

**Rationale:**

Kawasaki disease shock syndrome (KDSS) is a rare but severe complication of Kawasaki disease, often associated with intravenous immunoglobulin resistance, myocardial dysfunction, and higher risk of coronary complications. Leukemoid reaction is extremely uncommon in KDSS and can mimic hematologic malignancy, complicating timely diagnosis and management.

**Patient concerns:**

A 4-year-7-month-old boy presented with fever, cervical lymphadenopathy, rash, and hypotension. Laboratory findings showed extreme leukocytosis (peak white blood cell 71.10 × 10^9^/L), initially raising concern for acute leukemia.

**Diagnoses:**

The patient was diagnosed with KDSS based on fever, mucocutaneous features, circulatory collapse, multi-organ involvement, and echocardiographic findings. Bone marrow aspiration confirmed reactive hyperplasia without blasts, and additional workup excluded hematologic malignancy.

**Interventions:**

The child received intravenous immunoglobulin as first-line therapy but remained resistant. High-dose methylprednisolone was initiated, followed by tapering corticosteroids and low-dose aspirin. Short-term anticoagulation with dipyridamole and low-molecular-weight heparin was introduced as thromboprophylaxis, given the extreme inflammatory burden, hypoalbuminemia, serosal effusions, and elevated coagulation markers. Supportive therapy included albumin infusion, oxygen, and gastric protection.

**Outcomes:**

The patient showed rapid resolution of fever and shock after corticosteroid therapy, with progressive normalization of white blood cell and C-reactive protein levels. He was discharged in stable condition. At 1- and 24-month follow-up, laboratory results remained normal, and echocardiography confirmed absence of coronary artery aneurysms or ventricular dysfunction.

**Lessons:**

This case emphasizes that KDSS with leukemoid reaction may mimic hematologic malignancy, delaying appropriate immunomodulatory therapy. Extreme leukocytosis (≥70 × 10^9^/L) should not exclude KDSS, especially in children with unexplained fever and shock. Early recognition, adjunctive corticosteroid therapy, and individualized anticoagulation strategies are critical to prevent misdiagnosis and improve outcomes.

## 1. Introduction

Kawasaki disease (KD) is an acute, self-limiting systemic vasculitis predominantly affecting children under 5 years of age.^[1,2]^ Although most cases respond favorably to intravenous immunoglobulin (IVIG), a subset of patients develops Kawasaki disease shock syndrome (KDSS), a rare but life-threatening complication characterized by hypotension and signs of poor perfusion requiring intensive care support.^[3,4]^ KDSS accounts for approximately 1.4% to 7% of KD cases and is associated with higher risks of IVIG resistance, myocardial dysfunction, and coronary artery abnormalities.^[5]^ Its pathophysiology is thought to involve a cytokine storm and systemic capillary leak, contributing to multi-organ involvement and shock.^[6]^ The diagnosis remains primarily clinical, often delayed due to overlapping features with sepsis, toxic shock syndrome, and systemic juvenile idiopathic arthritis (sJIA).^[7]^

This case is particularly unique due to the presence of an extreme leukemoid reaction, with a white blood cell (WBC) count peaking at 71.10 × 10^9^/L. Such a degree of leukocytosis is uncommon in KDSS and can mimic hematologic malignancies, such as acute leukemia, leading to misdiagnosis or unnecessary invasive procedures.^[8]^ While moderate leukocytosis is frequently observed in KD or post-steroid administration,^[9]^ WBC counts exceeding 70 × 10^9^/L are exceedingly rare in nonmalignant pediatric cases. This case underscores the importance of differentiating reactive leukocytosis from malignant etiologies, especially in febrile children presenting with shock and lymphadenopathy. It also highlights the clinical utility of early corticosteroid intervention and supportive care in the management of IVIG-resistant KDSS complicated by leukemoid reaction.

## 2. Case

A 4-year and 7-month-old male presented with a 7-day history of a painful, enlarged left cervical lymph node (2.5 × 2.5 cm), followed by persistent fever (maximum 38.5°C) and a subsequent generalized rash. The patient had previously been hospitalized at a local facility where laboratory tests revealed leukocytosis (WBC 33.48 × 10^9^/L), neutrophilia (84.6%), and elevated C-reactive protein (CRP 92.65 mg/L). Despite initial treatment with IVIG and antibiotics, he continued to experience high fevers (up to 40.5°C), gastrointestinal symptoms, and progressive cervical lymphadenopathy. Upon transfer to the tertiary hospital, the patient exhibited bilateral conjunctival injection, extremity edema, mucosal changes, and cervical lymphadenopathy, fulfilling the criteria for KD. Clinical deterioration with hypotension, hypoalbuminemia, and serosal effusions led to a diagnosis of KDSS and IVIG resistance.

On admission, the physical examination revealed irritability, tachypnea, and 3-concave sign. Serial laboratory investigations showed a marked leukemoid reaction with WBC peaking at 71.10 × 10^9^/L on day 11, predominantly neutrophilic with the presence of metamyelocytes and myelocytes. CRP levels were initially elevated (166.31 mg/L), declining progressively with treatment. Blood gas analysis indicated hypoxemia and metabolic acidosis, while inflammatory cytokine profiling revealed significantly elevated IL-6 (346.6 pg/mL) and IL-10 (9.6 pg/mL), measured using enzyme-linked immunosorbent assay. These findings suggested a cytokine storm and supported our decision to escalate treatment with high-dose corticosteroids after IVIG resistance. Imaging studies demonstrated hepatomegaly, pleural effusions, ascites, cervical lymphadenopathy, and mild coronary artery dilation without aneurysms. Cardiac ultrasound showed mild tricuspid and mitral regurgitation, patent foramen ovale, and pulmonary hypertension. Given the extreme leukocytosis (>70 × 10^9^/L) and cervical lymphadenopathy, acute leukemia was initially suspected. Bone marrow aspiration was therefore performed and showed reactive hyperplasia without blasts. Peripheral smear and flow cytometry revealed no abnormal blast populations, further excluding hematologic malignancy. Additional infectious workup did not identify a bacterial source, and sepsis was considered less likely given the constellation of persistent fever, mucocutaneous features, and echocardiographic changes. Differential diagnoses such as sJIA and toxic shock syndrome were also evaluated, but the presence of shock, coronary involvement, and typical Kawasaki features ultimately supported the diagnosis of KDSS.

**Figure 1.**
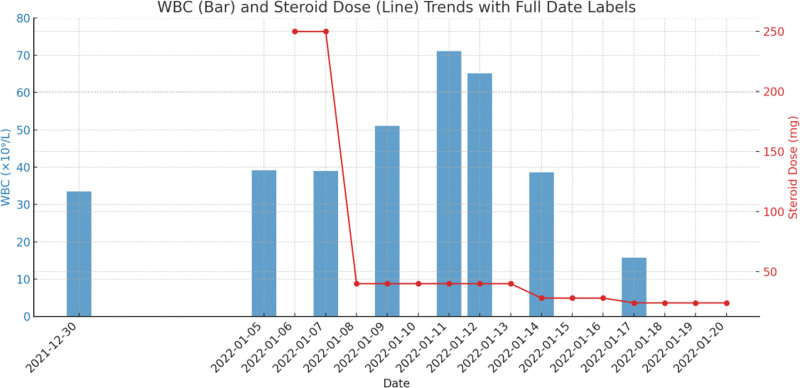
Trends in white blood cell count and steroid dosage over time. Temporal trends in white blood cell (WBC) count and corticosteroid administration in a pediatric patient with Kawasaki disease shock syndrome (KDSS) complicated by leukemoid reaction (peak WBC 71.10 × 10^9^/L). The WBC count (solid line) is plotted against the left y-axis (×10^9^/L), showing a sharp peak during acute disease progression. Concurrent intravenous methylprednisolone dosing (dashed line) is plotted against the right y-axis (mg/kg/day), indicating high-dose steroid initiation coinciding with leukocytosis and clinical deterioration. The graph highlights the dynamic interplay between inflammation and immunosuppressive therapy during the critical care phase. Date labels along the x-axis denote the clinical course timeline.

Due to poor response to initial IVIG therapy and progressive systemic inflammation, the patient was administered high-dose methylprednisolone (250 mg daily for 2 days), followed by tapering corticosteroids and low-dose aspirin. Anticoagulation was initiated with dipyridamole and low-molecular-weight heparin. as thromboprophylaxis. Although the patient had no coronary aneurysms, the decision was made based on the presence of Kawasaki disease shock syndrome with marked systemic inflammation, hypoalbuminemia, serosal effusions, and elevated coagulation markers, which are recognized risk factors for venous thromboembolism in critically ill children. This approach was consistent with individualized prophylaxis strategies described in pediatric critical care literature, while standard aspirin therapy was continued according to the American Heart Association guidelines.^[6,10–12]^ Supportive care included albumin infusion, oxygen therapy, and gastric protection. The patient’s condition gradually stabilized, with normalization of body temperature, reduction in WBC count (to 15.79 × 10^9^/L by day 17), and resolution of shock symptoms. He was discharged in stable condition with improved general appearance, resolution of lymphadenopathy, and desquamation on finger tips. Follow-up imaging confirmed clinical recovery without cardiac sequelae (Fig. 1; Table 1). At 1- and 24-month follow-up, laboratory tests demonstrated normalized white blood cell counts and CRP levels, and echocardiography consistently showed no evidence of coronary artery aneurysms or ventricular dysfunction.

**Table 1:** Clinical course and treatment summary of the current KDSS case with leukemoid reaction.

Date	Clinical course and treatment
January 5, 2024	Patient admitted with persistent fever, tachypnea, and signs of respiratory distress (notably 3-concave sign). Blood pressure monitored Q8H. Nasal oxygen administered. Initiated empiric antibiotic therapy with Ceftriaxone 1.4 g IV daily, and Aspirin 200 mg PO Q8H for antiplatelet therapy.
January 6, 2024	Persistent high fever with chills and transient hypotension. Laboratory results showed hypoalbuminemia; ultrasonography revealed polyserositis. Antibiotic regimen changed to Meropenem (Haizheng Meite) 0.3 g IV Q8H. Administered high-dose Methylprednisolone 0.25 g IV daily (01.06–01.07). Albumin 10 g IV daily and 100 mL normal saline rapid infusion were given for volume expansion. Bone marrow aspiration was performed for further evaluation. which revealed reactive hyperplasia without blasts, excluding acute leukemia. Additional workup, including peripheral smear, lactate dehydrogenase, uric acid, and cerebrospinal fluid cytology, showed no evidence of hematologic malignancy.
January 7, 2024	Lumbar puncture performed for cerebrospinal fluid analysis. Fever subsided starting from Day 3 post-admission.
January 8, 2024 to January 13, 2024	Continued tapering steroid therapy: Methylprednisolone 20mg IV BID. Clinical condition improved steadily.
January 11, 2024	Adjusted antiplatelet regimen: Aspirin reduced to 75 mg PO QD. Dipyridamole 25 mg PO TID added for enhanced anticoagulation support.
January 14, 2024 to January 16, 2024	Steroid tapering continued with Methylprednisolone 20 mg IV QM + Oral Medrol (Methylprednisolone) 8 mg QN.
January 17, 2024 to January 18, 2024	Further tapering: Methylprednisolone 20 mg IV QM + Medrol 4 mg PO QN.
January 19, 2024 to January 20, 2024	Oral steroid regimen: Medrol 20 mg PO QM + 4 mg PO QN.
January 5, 2024 to January 20, 2024	Supportive therapy throughout hospitalization included: • Omeprazole IV for gastric mucosa protection • Cardioprotective agents (Ruianji) • Nebulized therapy, alkalization, fluid/electrolyte correction, and calcium supplementation as needed
January 20, 2024	Patient clinically stable: afebrile, no rash, no joint pain, no vomiting or diarrhea. Discharged with physician approval.

BID = twice daily, IV = Intravenous, KDSS = Kawasaki Disease Shock Syndrome, PO = per os (oral administration), QD = once daily, QM = every morning, QN = every night, TID = three times daily, WBC = white blood cell count.

## 3. Discussion

KDSS is a severe, albeit rare, complication of KD, presenting with circulatory instability, multi-organ involvement, and increased inflammatory markers.^[13]^ Although KD typically affects younger children under 5 years, cases such as ours and several in the literature illustrate that KDSS may also occur in older children, often with more atypical or delayed manifestations.^[14]^ In our case, the child presented with a leukemoid reaction (WBC 71.10 × 10^9^/L), an extreme hematologic abnormality not commonly seen in KDSS. However, this finding was accompanied by markedly elevated inflammatory markers (CRP 166 mg/L, IL-6 346.6 pg/mL, IL-10 9.6 pg/mL) and imaging abnormalities including hepatomegaly, serosal effusions, and mild coronary artery dilation. These multimodal findings indicated a systemic hyperinflammatory response rather than a malignant process. While leukocytosis is typical in KD due to systemic inflammation, WBC counts above 50 × 10^9^/L raise significant concern for hematologic malignancy or paraneoplastic syndromes.^[15]^ Such cases often prompt unnecessary invasive diagnostic procedures such as bone marrow aspiration before KDSS is considered. Notably, Cheng et al reported a KDSS case presenting with cervical lymphadenitis and hypotension, initially misdiagnosed as sepsis.^[14]^ The presence of leukemoid reaction in our patient further amplified diagnostic complexity, underlining the necessity of clinical suspicion and timely recognition of KDSS in febrile children with atypical inflammatory responses (Table 2).

**Table 2:** Summary of published cases of KDSS.

Author	Year	Age	Sex	Diagnosis	WBC	Treatment
Nugud et al	2019	2 years	Female	Kawasaki disease shock syndrome (KDSS)	8.16 → 6.29 × 10^9^/L	IVIG 2 g/kg; no aspirin (due to influenza B); supportive care
Sinhabahu et al	2016	12 years	Female	Kawasaki shock syndrome (KSS)	5.58 → 20.15 × 10^9^/L	IVIG 2 g/kg; aspirin 80 mg/kg/day; ICU supportive care
Taddio et al	2016	Median 25 months	4 males, 1 female (KSS group)	Kawasaki shock syndrome (KSS)	Elevated, exact value not specified	IVIG; second dose or corticosteroids if resistant; vasoactive drugs; ICU care
Tissandier et al	2014	6 years	Male	KSS with recurrent incomplete KD	18.53 × 10^9^/L	IVIG 2 g/kg over 2 days; methylprednisolone 30 mg/kg/day for 3 days; aspirin; diuretics

ICU = intensive care unit, IVIG = intravenous immunoglobulin, KD = Kawasaki disease, KDSS = Kawasaki disease shock syndrome, KSS = Kawasaki shock syndrome, WBC = white blood cell count.

A review of published cases demonstrates a consistent association between KDSS and more severe systemic inflammation, IVIG resistance, and frequent cardiac involvement.^[16,17]^ Taddio et al found KDSS patients had significantly higher CRP, lower hemoglobin, and hypoalbuminemia compared to typical KD cases, with 60% of KDSS patients showing IVIG resistance.^[16]^ Similarly, in our case, initial administration of IVIG failed to induce clinical improvement, indicating IVIG resistance; the patient subsequently responded well to adjunctive corticosteroid therapy and comprehensive supportive care. The decision to initiate corticosteroids was further supported by cytokine profiling, which demonstrated markedly elevated IL-6 and IL-10 consistent with hyperinflammation. While the American Heart Association recommends IVIG as first-line therapy, recent reports highlight the benefit of adjunct corticosteroids in severe or IVIG-resistant KDSS.^[18–20]^ In accordance with the 2017 and 2024 AHA guidelines, aspirin is recommended for all patients with KD, and systemic anticoagulation is generally reserved for those with giant coronary artery aneurysms or thrombosis. In our case, anticoagulation was introduced not for coronary involvement but as a short-term thromboprophylaxis strategy, given the extreme inflammatory burden and multiple prothrombotic risk factors associated with KDSS. Similar individualized decisions have been reported in pediatric critical care settings where venous thromboembolism prophylaxis is considered for high-risk patients.^[6,10–12]^ For instance, Tissandier et al successfully treated a recurrent KDSS case with IVIG and high-dose methylprednisolone, resulting in rapid clinical recovery and normalization of ventricular function.^[17]^ In our case, corticosteroid therapy likely contributed to the prompt resolution of fever and hemodynamic instability after IVIG failure, by mitigating the ongoing hyperinflammatory response. In retrospect, earlier initiation of corticosteroids might have shortened the disease course and reduced systemic complications, which is consistent with emerging evidence supporting early adjunctive corticosteroid therapy in IVIG-resistant or severe KDSS cases.^[21,22]^ The initial diagnostic challenge, amplified by the extreme leukocytosis, underscores the importance of early recognition of KDSS, even in the presence of features mimicking hematologic malignancy. Comparative analyses with previously published KDSS cases further contextualize our findings. Chen et al reported that KDSS patients had significantly higher CRP levels, neutrophil counts, and IVIG resistance compared to non-shock KD cases, consistent with our patient’s inflammatory profile and treatment response.^[23]^ Li et al described 27 KDSS patients with almost universal corticosteroid use, hemodynamic instability, and higher coronary involvement compared with typical KD, again aligning with our patient’s need for adjunctive corticosteroids but with the notable absence of coronary sequelae.^[6]^ Other severe phenotypes, such as KDSS complicated with ARDS requiring ECMO (Liu et al) and KDSS with MAS (Yi et al), illustrate the clinical variability and complexity of this syndrome.^[24]^ Compared with these reports, our case underscores an unusual hematologic presentation, extreme leukemoid reaction, yet with favorable recovery under timely corticosteroid therapy.

This case adds to the growing literature highlighting the atypical and severe spectrum of KDSS. Importantly, the constellation of extreme leukocytosis (71.10 × 10^9^/L), cytokine storm profile (IL-6/IL-10 elevation), hypoalbuminemia, and echocardiographic changes underscores that KDSS should be recognized as a systemic inflammatory syndrome rather than interpreted solely through hematologic findings. While the leukemoid reaction mimicked leukemia and thus risked mismanagement, integration of biomarker and imaging evidence provided crucial guidance for timely diagnosis and treatment. In the published literature, KDSS cases explicitly documenting a leukemoid reaction are scarce. As summarized in Table 2, most reported WBC peaks are lower than in our patient; his peak of 71.10 × 10^9^/L therefore appears to lie at the extreme end of the reported spectrum, highlighting the diagnostic challenge and the importance of considering KDSS in the differential diagnosis of febrile children presenting with shock and marked leukocytosis (Table 2). While leukemoid reactions have been described in association with severe infections and inflammatory syndromes, including HLH and sJIA, reports of such hematologic responses in KDSS remain exceedingly rare.^[25–27]^ In addition, whether the leukemoid reaction in this case represents a distinct immunopathologic phenotype of KDSS or simply an extreme inflammatory response deserves consideration. Studies show that KDSS patients often display neutrophilia, leukocytosis, elevated cytokine levels (IL-6, IL-10), hypoalbuminemia, and IVIG resistance, features that align with exaggerated systemic inflammation rather than a fundamentally different phenotype. However, immunophenotyping work (see “Immunopathogenesis of Kawasaki-Like Diseases” and “Immunophenotype of Kawasaki Disease” reviews) suggests that in some KDSS cases there may be a more pronounced activation of innate immune cells, dysregulated cytokine networks, and potentially genetic or molecular differences that influence the severity and treatment response. Thus, this leukemoid reaction may lie on a spectrum: at one end, an extreme inflammatory response to KDSS; at the other, a rare, severe immune dysregulation subtype, more case reports and immune profiling will be needed to distinguish between them.^[28,29]^ Sinhabahu et al also reported a KDSS case initially managed as septic shock, later diagnosed based on evolving clinical and echocardiographic features.^[15]^ Notably, our case showed good recovery without coronary artery complications, likely due to timely corticosteroid use, volume support, and close monitoring. Lessons from this case emphasize 3 diagnostic pitfalls in KDSS: atypical age, absence of initial full diagnostic criteria, and hematologic features mimicking malignancy. Recognizing these features can reduce misdiagnosis and prevent delays in immunomodulatory therapy. A high index of suspicion and awareness of atypical KDSS presentations, especially in children with unexplained fever, shock, and leukocytosis, are paramount in improving outcomes.

This study has some limitations. First, it represents a single case report, and therefore the generalizability of the findings is limited. Larger case series and multi-center studies will be needed to confirm whether the observations described here apply to the broader pediatric population. Second, long-term follow-up beyond 2 years was not available through our institution, although telephone consultation with the parents indicated that the child remained stable without recurrence. Despite these limitations, this report adds valuable clinical evidence by documenting an extreme leukemoid reaction in KDSS, a presentation rarely reported in the literature.

This case underscores the importance of recognizing KDSS in children with unexplained shock and extreme leukocytosis. A WBC count as high as 71.10 × 10^9^/L can mimic leukemia, leading to misdiagnosis. Early identification and timely immunomodulatory therapy, especially in IVIG-resistant cases, are essential to avoid complications and ensure recovery.

## Acknowledgments

We thank the patient who accepted to participate in this study.

## Author contributions

**Conceptualization:** Xiaoxian Wang.

**Writing – original draft:** Lingke Liu.

**Writing – review & editing:** Lingke Liu, Wenjie Xuan, Yana Wang, Nan Tu, Boting Pan.

## References

[1] Rife E Gedalia A. Kawasaki disease: an update. Curr Rheumatol Rep. 2020;22:75. 32924089 10.1007/s11926-020-00941-4 PMC7487199

[2] Day-Lewis M Son MBF Lo MS. Kawasaki disease: contemporary perspectives. Lancet Child Adolesc Health. 2024;8:781–92. 39299749 10.1016/S2352-4642(24)00169-X

[3] Zhao Z Yuan Y Gao L Li Q Wang Y Zhao S. Predicting Kawasaki disease shock syndrome in children. Front Immunol. 2024;15:1400046. 38887295 10.3389/fimmu.2024.1400046 PMC11180713

[4] Bejiqi R Pajaziti N Agushi S. Kawasaki disease shock syndrome presented with giant coronary artery dilatation - presentation of two cases and a literature review. Acta Inform Med. 2022;30:253–6. 36311151 10.5455/aim.2022.30.253-256 PMC9559664

[5] Gamez-Gonzalez LB Moribe-Quintero I Cisneros-Castolo M. Kawasaki disease shock syndrome: unique and severe subtype of Kawasaki disease. Pediatr Int. 2018;60:781–90. 29888440 10.1111/ped.13614

[6] Li Y Zheng Q Zou L. Kawasaki disease shock syndrome: clinical characteristics and possible use of IL-6, IL-10 and IFN-γ as biomarkers for early recognition. Pediatr Rheumatol Online J. 2019;17:1. 30611297 10.1186/s12969-018-0303-4 PMC6321686

[7] Tsai HY Lee JH Yu HH Wang LC Yang YH Chiang BL. Initial manifestations and clinical course of systemic onset juvenile idiopathic arthritis: a ten-year retrospective study. J Formos Med Assoc. 2012;111:542–9. 23089689 10.1016/j.jfma.2011.06.013

[8] Matsuzaki K Okumi M Kishimoto N. A case of bladder cancer producing granulocyte colony-stimulating factor and interleukin-6 causing respiratory failure treated with neoadjuvant systemic chemotherapy along with sivelestat. Hinyokika Kiyo. 2013;59:443–7. 23945326

[9] Hu SC Chen GS Chen PH Chiou SS Lan CC. Leukocytosis associated with low leukocyte alkaline phosphatase score following intravenous steroid therapy for atopic dermatitis. J Eur Acad Dermatol Venereol. 2008;22:1266–7. 18341533 10.1111/j.1468-3083.2008.02618.x

[10] McCrindle BW Rowley AH Newburger JW. Diagnosis, treatment, and long-term management of Kawasaki disease: a scientific statement for health professionals from the American Heart Association. Circulation. 2017;135:e927–99. 28356445 10.1161/CIR.0000000000000484

[11] Jone PN Tremoulet A Choueiter N. Update on diagnosis and management of Kawasaki disease: a scientific statement from the American Heart Association. Circulation. 2024;150:e481–500. 39534969 10.1161/CIR.0000000000001295

[12] Li B Liu X Shao S. Predictive value of coagulation profiles for Kawasaki disease shock syndrome: a prospective cohort study. Front Pediatr. 2024;12:1450710. 39220153 10.3389/fped.2024.1450710 PMC11362036

[13] Power A Runeckles K Manlhiot C Dragulescu A Guerguerian AM McCrindle BW. Kawasaki disease shock syndrome versus septic shock: early differentiating features despite overlapping clinical profiles. J Pediatr. 2021;231:162–7. 33290811 10.1016/j.jpeds.2020.12.002

[14] Cheng YT Lee YS Lin JJ Chung HT Huang YC Su KW. Kawasaki shock syndrome with initial presentation as neck lymphadenitis: a case report. Children (Basel). 2022;9:56. 35053681 10.3390/children9010056 PMC8774107

[15] Sinhabahu VP Suntharesan J Wijesekara DS. Kawasaki shock syndrome in a 12-year-old girl mimicking septic shock. Case Rep Infect Dis. 2016;2016:4949036. 28101385 10.1155/2016/4949036 PMC5215141

[16] Taddio A Rossi ED Monasta L. Describing Kawasaki shock syndrome: results from a retrospective study and literature review. Clin Rheumatol. 2017;36:223–8. 27230223 10.1007/s10067-016-3316-8

[17] Tissandier C Lang M Lusson JR Bœuf B Merlin E Dauphin C. Kawasaki shock syndrome complicating a recurrence of Kawasaki disease. Pediatrics. 2014;134:e1695–1699. 25384485 10.1542/peds.2014-0004

[18] Zheng Z Huang Y Wang Z. Clinical features in children with Kawasaki disease shock syndrome: a systematic review and meta-analysis. Front Cardiovasc Med. 2021;8:736352. 34621802 10.3389/fcvm.2021.736352 PMC8491834

[19] Lin Y Shi L Deng YJ Liu Y Zhang HW. Kawasaki disease shock syndrome complicated with macrophage activation syndrome in a 5-month old boy: a case report. Medicine (Baltimore). 2019;98:e14203. 30681594 10.1097/MD.0000000000014203 PMC6358384

[20] Sato T Somura J Maruo Y. Steroid pulse therapy for Kawasaki disease complicated with myocarditis. Indian Pediatr. 2016;53:1015–6. 27889733 10.1007/s13312-016-0979-9

[21] Shulman ST. Early steroid therapy reduces Kawasaki disease coronary complications. J Pediatr. 2017;182:401–4. 10.1016/j.jpeds.2016.12.065 28237455

[22] Marchesi A Recupero R Sardella L. The protective role of steroids on coronary arteries in the acute phase of Kawasaki disease for high risk patients: a retrospective study. Front Pediatr. 2025;13:1636339. 40948495 10.3389/fped.2025.1636339 PMC12424431

[23] Chen PS Chi H Huang FY Peng CC Chen MR Chiu NC. Clinical manifestations of Kawasaki disease shock syndrome: a case-control study. J Microbiol Immunol Infect. 2015;48:43–50. 23927822 10.1016/j.jmii.2013.06.005

[24] Liu J Yang C Zhang Z Li Y. Kawasaki disease shock syndrome with acute respiratory distress syndrome in a child: a case report and literature review. BMC Pulm Med. 2022;22:220. 35668424 10.1186/s12890-022-02007-w PMC9168351

[25] Naaraayan A Aleta M Basak P Jesmajian S Goldstein R. Leukemoid reaction to Clostridium difficile infection. Anaerobe. 2015;34:158–60. 25978982 10.1016/j.anaerobe.2015.05.005

[26] Tarekegn K Colon Ramos A Sequeira Gross HG Yu M Fulger I. Leukemoid reaction in a patient with severe COVID-19 infection. Cureus. 2021;13:e13598. 33815998 10.7759/cureus.13598 PMC8011462

[27] Feng MT Ji Q Liu DD Xu W. Two cases of Leukemoid reaction in premature infants caused by fetal inflammatory response syndrome. BMC Pediatr. 2024;24:546. 39182037 10.1186/s12887-024-05006-4 PMC11344429

[28] Chen MR Kuo HC Lee YJ. Phenotype, susceptibility, autoimmunity, and immunotherapy between Kawasaki disease and coronavirus disease-19 associated multisystem inflammatory syndrome in children. Front Immunol. 2021;12:632890. 33732254 10.3389/fimmu.2021.632890 PMC7959769

[29] Agrafiotou A Sapountzi E Margoni A Fotis L. Immunophenotype of Kawasaki disease: insights into pathogenesis and treatment response. Life (Basel). 2025;15:1012. 40724515 10.3390/life15071012 PMC12299537

